# Discontinuation of Pegvaliase therapy during maternal PKU pregnancy and postnatal breastfeeding: A case report

**DOI:** 10.1016/j.ymgmr.2019.100555

**Published:** 2020-01-10

**Authors:** Fran Rohr, Amy Kritzer, Cary O. Harding, Krista Viau, Harvey L. Levy

**Affiliations:** a Boston Children's Hospital, 300 Longwood Ave, Boston, MA 02115, United States of America; b Met Ed Co, Boulder, CO, 80302, United States of America; c Department of Pediatrics, Harvard Medical School, Boston, MA, 02115, United States of America; d Oregon Health and Science University, Portland, OR 97239, United States of America

**Keywords:** PKU, Phenylketonuria, Maternal PKU, Breastfeeding, Pegvaliase, Phenylalanine ammonia lyase

## 1 Introduction

Phenylketonuria (PKU) is an inherited metabolic disorder due to phenylalanine hydroxylase (PAH) deficiency resulting in an elevation of Phe [1]. High blood phenylalanine (Phe) is associated with developmental disability, seizures and eczema that are prevented if blood Phe is controlled from infancy within 120–360 μmol/L [2]. The primary treatment for PKU is a Phe-restricted diet that eliminates high protein food and relies on the use of low or Phe-free medical foods. The diet is difficult to follow, and this results in sub-optimal blood Phe, especially in adults [3], with associated adverse neuropsychological outcomes [4]. Consequently, additional non-dietary therapies have been developed. The first of these to be approved was saproterin dihydrochloride,^1^ a synthetic form of the PAH cofactor tetrahydrobiopterin (BH_4_). In pharmacological amounts this oral therapy usually serves as adjunctive to diet by enhancing PAH activity in a subpopulation of those with PKU, thereby allowing for a degree of diet liberalization in those patients [5]. A second and more recently approved therapy is pegvaliase pqpz^1^ (pegvaliase), an injectable enzyme substitution therapy that converts phenylalanine to trans-cinnamic acid and ammonia and can replace dietary therapy in those with PKU who respond to this treatment [6].

Untreated PKU during pregnancy (maternal PKU) results in poor offspring outcomes, including low birth weight, microcephaly, congenital heart defects and developmental disability [7,8]. During clinical trials of pegvaliase, subjects were required to use two forms of birth control and to discontinue the study drug for pregnancy and breastfeeding but the approved label in the U.S. does not contraindicate its use during pregnancy or breastfeeding [9]. Therefore, this decision is left to the judgment of prescribing clinicians. However, there are no human data available regarding the use of pegvaliase during pregnancy or breastfeeding.

This case describes the challenges for a woman with PKU who transitioned from pegvaliase therapy and an unrestricted diet to a phe-restricted diet for pregnancy and breastfeeding and the re-introduction of drug therapy after breastfeeding.

## 2 Case report

This is a woman with classic PKU [PAH genotype R408W/842 + 3G > C] identified through newborn screening and started on a Phe-restricted diet at age 10 days. Dietary Phe tolerance was 175 mg/d at age 3 years. Blood Phe was well-controlled through childhood but after age 18 years her blood Phe began to increase, ranging up to 1020 umol/L. The highest blood Phe concentrations corresponded to a period during college when she had a higher Phe intake leading to therapy with large neutral amino acids. At age 24 years she was given a trial of sapropterin, but there was no reduction in blood Phe and she remained on a Phe-restricted diet. At age 27 years she enrolled in the PRISM-2 pegvaliase clinical trial and was randomized to receive a dose of 40 mg/d. Her blood Phe decreased from 914 μmol/L to 9 μmol/L after 13 weeks on pegvaliase; side effects during this time included mild to moderate arthralgia and skin reactions, both of which resolved. Her response to pegvaliase allowed her to transition to a normal diet over a 6-week period, including an intact protein intake of 75–80 g/d and elimination of medical food while her blood Phe remained low (<30 μmol/L). Subsequently, her blood Phe increased to high concentrations and the pegvaliase dose was increased to 60 mg/d. She had been on this dose for 19 months when at age 31 years she decided to discontinue therapy and plan a maternal PKU pregnancy.

The goals for pregnancy included maintaining blood Phe between 120 and 360 μmol/L for one month while off pegvaliase before discontinuing birth control and concurrently consuming at least 70 g/d of total protein as well as meeting other nutrient requirements for pregnancy. This was achieved by slowly reducing the dose of pegvaliase while reinstituting the Phe-restricted diet. The patient, who is a registered dietitian, collaborated with the metabolic dietitians in developing a -step transition plan (Table 1). In the first step, intact protein decreased by 30 g and medical food was increased by 30 g, thereafter each step had approximately 10 g less intact protein and 10 g more medical food protein. As the Phe-diet was re-introduced, all Phe from foods were counted with a limit of 175 mg/d, the patient's blood Phe tolerance as a child.

**Table 1:** Nutrition plan for transition off pegvaliase and a normal diet to a Phe-restricted diet.

Week	Pegvaliase dose (mg/d)	Protein intact (g)^a^	Protein medical food (g)	Total protein (g)	Phe from food^b^ (mg)
Baseline	60	75	0	75	175
Week 1–2	40	44	35	79	175
Week 3–4	40	33	35	68	175
Week 5–6	20	22	55	77	175
Week 7–8	20	11	55	66	175
Week 9–10	0	0	75	75	175

a Provided as whey protein (1 scoop = 11 g protein) mixed into smoothies starting Week 1.

b Phe from food contributed approximately 3 g of low-quality protein that is not included in the total protein intake.

**Fig. 1:**
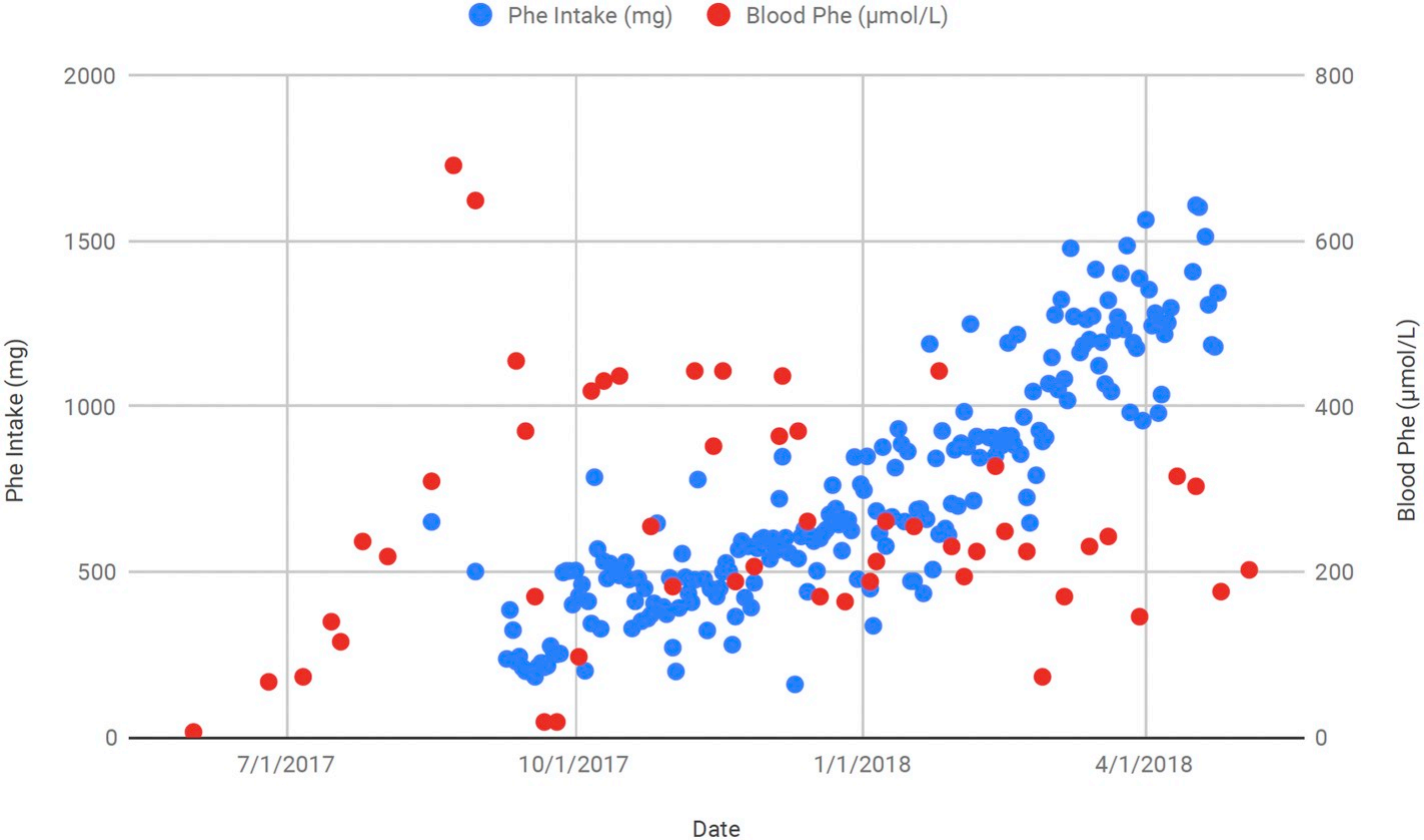
Blood Phe (μmol/L) (red) and Phe intake (mg/d) (blue) during transition off pegvaliase and during in a woman with PKU. (For interpretation of the references to color in this figure legend, the reader is referred to the web version of this article.)

Fig. 1 shows intake of Phe and blood Phe during the transition period off pegvaliase and throughout pregnancy. Pregnancy occurred 4 weeks after pegvaliase was fully discontinued and mean blood Phe during pregnancy was 300 ± 150 umol/L. Pregnancy was complicated by gestational diabetes requiring insulin. A female infant was born at 39 weeks' gestation, weighing 3202 kg (50th percentile) and measuring 50.8 cm in length (82nd percentile) and 33.5 cm in head circumference (38th percentile) [10]. She is currently 18 months old and developing normally.

The patient remained on a Phe-restricted diet during breastfeeding for 7 months during which time her blood Phe increased from 624 to 1406 μmol/L. When the baby was weaned the mother restarted pegvaliase therapy with an accelerated re-introduction schedule (Table 2). Blood Phe declined within 6 weeks to below normal concentrations so intact protein was reintroduced over a 2-week period to approximately 80 g/d. The patient was counseled to eat normally without counting protein intake. Blood Phe then increased to 309 and 370 μmol/L and the pegvaliase dose was increased to 30 mg/d. Blood Phe concentrations again were low and her dose was decreased to 30 mg on Mon, Wed, Friday and 20 mg on Tues, Thurs, Sat and Sunday (Table 2). She has remained on that regimen.

**Table 2:** Titration schedule and blood Phe during reintroduction of pegvaliase after pregnancy and breastfeeding in woman with PKU.

Week	Pegvaliase dose (mg)	Frequency	Blood Phe (μmol/L)
1	2.5	2× weekly	1406
2	10	1× weekly	1230
3	10	2× weekly	Not done
4	10	4× weekly	1484
5	10	Daily	Not done
6	20	Daily	648
7	20	Daily	30
8	20	Daily	36
11	20	Daily	109
12	20	Daily	309
14	20	Daily	370
19	20	Daily	48
21	20	Daily	170
23	30	Daily	246
25	30	Mon, Wed, Fri	6
	20	Sun, Tue, Thr, Sat	
27	30	Mon, Wed, Fri	0
	20	Sun, Tue, Thr, Sat	
32	30	Mon, Wed, Fri	18
	20	Sun, Tue, Thr, Sat	
33	30	Mon, Wed, Fri	36
	20	Sun, Tue, Thr, Sat	

After pegvaliase was re-introduced, the patient pumped her breast milk to obtain samples for analysis of pegvaliase content. The breast milk was kept in a non-frost-free freezer until shipped frozen on dry ice to the Harding Lab at OHSU for assessment of phenylalanine ammonia lyase (PAL). PAL activity was measured using a spectrophotometric method to detect the production of *trans*-cinnamic acid at 290 nM following the incubation of a skimmed milk aliquot with 13 mM *l*-phenylalanine in 100 mM TRIS buffer, pH 8.0 at 37.0 °C. PAL activity was also measured in a control breast milk sample collected from a woman without PKU. The spectrophotometric assay used was reliably able to detect a minimum production of 1 nmoL *trans*-cinnamic acid per min. The measured PAL activity in the breast milk sample from the pegvaliase treated PKU patient (0.15 ± 0.24 nmol/min) was indistinguishable from that measured in TRIS buffer blanks (0.02 ± 0.03) or the control breast milk sample (0.47 ± 0.52) and was essentially below the lowest reliable limit of detection for the assay. To control for the possibility of any anti-pegvaliase inhibitors in the milk, the assay was repeated after spiking the milk with commercially available pegvaliase (20 mg/mL, 10 μL added to 1 mL skim breast milk). The measured PAL activity of added pegvaliase was similar in both breast milk from the pegvaliase-treated patient and in the control milk sample to the measured activity in TRIS buffer indicating the absence of any inhibitors of pegvaliase activity in the milk samples.

## 3 Discussion

There is limited guidance about discontinuing pegvaliase for pregnancy and resuming therapy afterward. For discontinuing pegvaliase we developed a 10-week transition plan aimed at providing approximately 70 g/d of total protein while slowly decreasing the drug and intact protein and increasing medical food protein intake. The goal was to prevent an increase in blood Phe. We were concerned about high blood Phe interfering with neurocognition, which in turn would impact the patient's ability to strictly follow the Phe-restricted diet. Due to our observations that some patients in the clinical trials who had discontinued pegvaliase abruptly reported having headaches, lethargy and malaise, we chose to discontinue the drug very slowly. In retrospect, while the 2-week period for each step in the transition was successful in this case, a 1-week period could be tried for pregnancies to reduce the length of transition period. The patient observed that the transition from an unrestricted diet to a very limited diet and medical foods was intimidating. However, compared to other times in her life when her blood Phe was high, following the diet while having blood Phe in good control was easier, likely due to improved executive function and less anxiety.

The patient chose to eliminate all high protein foods as soon as the pegvaliase dose was first decreased from 60 to 40 mg/d. She felt this would be easier than slowly eliminating high protein foods in her diet and having to count protein intake. Thus, her protein intake consisted of intact protein supplied as whey protein in smoothies along with medical food protein from formula, and a limited amount of Phe from food (175 mg). Once she was completely off pegvaliase, her blood Phe remained low, indicating that her Phe tolerance was higher than 175 mg or the pegvaliase had not completely cleared from her system. Pegvaliase should be eliminated from body tissues within 4 weeks of discontinuation [11]. Others have reported that Phe tolerance in adults is often higher than during childhood and needs to be re-evaluated [12]. After the transition off pegvaliase, the patient slowly increased her Phe intake to 400–500 mg/d while maintaining blood Phe between 120 and 360 μmol/L. One month later she became pregnant. Once pregnant, the patient noted that compliance was much easier stimulated by the knowledge that well-being of the baby depended on tight control of blood Phe. Additionally, the intact protein allowance rapidly increased throughout gestation to approximately 30 g/d, so following the diet was more manageable.

After pregnancy, the patient was eager to resume pegvaliase and questioned if this was safe for a breastfed infant. Since no information was available on the use of pegvaliase during breastfeeding, the patient was advised to not begin the drug while breastfeeding although there was conflicting opinion on this advice, at least two of us believing that breastfeeding while on pegvaliase was likely to be safe for the infant. Whether pegvaliase would be present in breast milk was unknown and even if present its absorption by the infant from breast milk seemed unlikely given that it is a polypeptide that is probably destroyed in the infant's gastrointestinal tract. Nevertheless, there remained the possibility that decreased Phe in the mother might decrease the Phe content of breast milk [13]. Additionally, patients starting pegvaliase treatment require premedication with both Zyrtec and Zantac because of immunogenicity of the drug. Thus, when deciding whether to be on a drug during breastfeeding, many factors need to be considered including whether there are other therapeutic alternatives for the mother, the impact of the drug on milk production, the amount of drug excreted into breast milk and the potential impact of the drug on the breastfeeding infant [14].

The advice to withhold pegvaliase during breastfeeding was based on the uncertainty of its safety given the high immunogenicity of pegvaliase and its unknown impact on the quantity or quality of breast milk compared to the known safety of breastfeeding while on a Phe-restricted diet [15]. Additional consideration was given to the fact that adverse events associated with drug exposure during breastfeeding occur most often in neonates younger than 2 months of age [16]. Moreover, hypophenylalaninemia, a potential complication of pegvaliase therapy, could result in low breast milk Phe and hypophenylalaninemia in the breastfeeding infant, potentially producing gastrointestinal, dermatological and ocular complications [17]. Nevertheless, it is possible that none of these concerns would occur, that breastfeeding while on pegvaliase is harmless.

The costs of continuing a Phe-restricted diet versus resuming pegvaliase during breastfeeding must be considered as well. These include the difficulty in maintaining a strict diet and the likelihood of blood Phe increasing above therapeutic goals. In a study of pregnant women, even women who do well on a Phe-restricted diet during pregnancy have more difficulty adhering to the diet after childbirth [18]. This was observed in our patient who sent 4 blood spots (mean 721 μmol/L) in the 2 months after delivery; the next blood phe was obtained 1406 μmol/L when she came to clinic to restart pegvaliase. High blood Phe in mothers with PKU after childbirth can result in neurocognitive deficits that affect parenting and is associated with a higher incidence of depression (by self-report) [19]. In addition, while the Phe-restricted diet is adequate to support breastfeeding, it is a semi-synthetic diet and the micronutrient content of the breast milk of mothers with PKU is not known.

The patient observed that the transition back to a very low intact protein diet in the post-partum period was particularly challenging because of the lack of time and energy to plan meals and cook, as well as trying to meet the energy demands of breastfeeding with very limited food choices, While the patient had intended to breastfeed for one year, when faced with the emotionally difficult decision to be on the Phe-restricted diet or stop breastfeeding, she decided to wean the baby earlier than anticipated and restart pegvaliase when her baby was 7 months old.

We found no evidence that pegvaliase activity, which circulates in blood of pegvaliase-treated PKU patients, penetrated into breast milk in this single case, at least at the pegvaliase dose of 20 mg/day in the mother. We did not analyze the breast milk for the presence of phenylalanine ammonia lyase (PAL) protein using immunologic methods. Even if minor amounts of PAL protein are secreted in milk, these would be proteolyzed by the infant digestive tract and would not survive to affect the infant's Phe intake. Given that PAL is active against only free Phe and that the Phe intake in a breastfed infant is mediated via intact protein, predominantly as lactalbumin, any trace amount of undetected PAL activity in breast milk will not alter infant nutrition. Based upon this result and our understanding of breast milk production biology and pegvaliase pharmacodynamics, our professional opinion is that breast feeding should not be contraindicated during pegvaliase treatment. Therefore, in subsequent pregnancies that we follow, pegvaliase therapy would be restarted as soon as possible in the postpartum period, even in a mother who was breastfeeding her infant, in order to minimize the likelihood of the mother's blood Phe becoming elevated.

After the patient discontinued breastfeeding and pegvaliase was introduced, she did well with the accelerated dosing regimen (Table 2). This was expected since during the clinical trials, following discontinuation of pegvaliase for the randomized withdrawal period, the subjects tolerated the return to their usual dose within a week. Even patients who have discontinued pegvaliase due to anaphylactoid-like reactions, have successfully returned to therapy under close observation [11]. While a dose of 60 mg/d was required to maintain blood Phe in the target range prior to pregnancy in our patient, she exhibited a response at a much lower dose of 20 mg/d when pegvaliase therapy was restarted, as has been our experience when restarting therapy in other individuals. Intact protein was also reintroduced rapidly in our patient because after she had responded to pegvaliase during the clinical trials, she had become accustomed to eating high protein foods and had maintained blood Phe in treatment range (or below) on a normal protein intake. Thus, the suggested 10–20 g increments in protein intake [11] may not be necessary for patients returning to drug therapy.

In conclusion, this case represents one of the few patients who have discontinued pegvaliase for pregnancy and breastfeeding and indicates the clinical decisions that must be made about how to transition off pegvaliase and return to a Phe-restricted diet, as well as when and how quickly to reintroduce the drug and a normal diet in patients wanting to resume pegvaliase. It also highlights the psychosocial impact of these transitions on the patient.

## Funding source

This research did not receive any specific grant from funding agencies in the public, commercial, or not-for-profit sectors.

## Footnotes

1 Biomarin Pharmaceutical Inc., Novato, CA 94949.
